# Notices of New Publications

**Published:** 1873-06

**Authors:** 


### Notices of New Publications.

*The Obstetrical Journal of Great Britain and Ireland* is the title of a new monthly reprint, of which the first number was issued in April. Its English editors are Drs. James H. Aveling and Alfred Wiltshire, who are supported by a large corps of prominent British obstetricians and surgeons. It is published in Philadelphia by Henry C. Lea, with an American supplement edited by William F. Jenks, M. D. The present number contains seventy-two pages with sixteen pages additional in the American part. It is furnished at five dollars per year, is printed in the best style and will be a welcome and desirable addition to the literature of the department. It contains interesting articles by Dr. Barnes and other well-known writers.——*The Sanitarian*, a monthly journal of ninety-six pages, commenced in May, is edited by Dr. A. N. Bell, of Brooklyn, and published by A. S. Barnes & Co., New York. Its purpose is to present the results of the various inquiries which have been and which may hereafter be made for the preservation of health and the expectations of human life, as to make them most advantageous to the public and the medical profession. The present number contains very interesting and valuable articles by Moreau Morris, M. D., Jerome Walker, M. D., J. M. Toner, M. D., M. D., by the editor, Dr. Bell, who has heretofore furnished articles for our own pages, and by William A. Hammond, M. D., on the Sanitary Influence of Light, an extract from which our readers will find elsewhere in the

Journal. It is published at three dollars a year, in advance, and will be subscribed for by all who take special interest in this department.——The *Charleston Medical Journal and Review* is a quarterly journal of eighty-eight pages, commenced in April, under the editorship of E. Peyre Porcher, M. D., Professor of Clinical Medicine in the Medical College at Charleston, S. C, and by R. A. Kinlock, M. D., Professor of Surgery in the same institution. It is the revival of the old Charleston *Medical Journal* under promising auspices; its first issue has a good number of interesting original articles, and no doubt the publication will meet with extended favor which it really deserves — -The *Herald of Health*, published by Wood & Holbrook, of New York, has published its June number, which contains a great number of interesting articles on popular subjects. Each number contains forty pages, and the publi.ation is furnished for the low price of $1 50 per year.

